# Postoperative complications and surgical outcomes of robotic *versus* conventional nipple-sparing mastectomy in breast cancer: meta-analysis

**DOI:** 10.1093/bjs/znad336

**Published:** 2023-10-27

**Authors:** Ashrafun Nessa, Shafaque Shaikh, Mairi Fuller, Yazan A Masannat, Stavroula L Kastora

**Affiliations:** School of Medicine, Medical Sciences and Nutrition, University of Aberdeen, Aberdeen, UK; General Surgery, Aberdeen Royal Infirmary, Aberdeen, UK; Breast Surgery, Aberdeen Royal Infirmary, Aberdeen, UK; School of Medicine, Medical Sciences and Nutrition, University of Aberdeen, Aberdeen, UK; General Surgery, Aberdeen Royal Infirmary, Aberdeen, UK; Breast Surgery, Aberdeen Royal Infirmary, Aberdeen, UK; School of Medicine, Medical Sciences and Nutrition, University of Aberdeen, Aberdeen, UK; Breast Surgery, Aberdeen Royal Infirmary, Aberdeen, UK; UCL EGA Institute for Women's Health, University College London, London, UK

## Abstract

**Background:**

Breast cancer is the most common cancer worldwide, with remarkable advances in early diagnosis, systemic treatments, and surgical techniques. Robotic nipple-sparing mastectomy has been trialled; however, the complication rates, surgical outcomes, and oncological safety of this approach remain obscure.

**Methods:**

A systematic search of the literature was conducted from conception until September 2022. Studies examining complications and operative variables where robotic nipple-sparing mastectomy was compared with conventional nipple-sparing mastectomy were included. Primary study outcomes were complications (Clavien–Dindo grade III complications, skin or nipple necrosis, seroma, haematoma, infection, implant loss, and wound dehiscence) and oncological safety (recurrence and positive margins). The secondary outcomes included operative variables, length of stay, cost-effectiveness, learning curve, and aesthetic outcome.

**Results:**

A total of seven studies of overall fair quality, involving 1674 patients, were included in the systematic review and meta-analysis. Grade 3 complications were reduced in robotic nipple-sparing mastectomy without statistical significance (OR 0.60 (95 per cent c.i. 0.35 to 1.05)). Nipple necrosis was significantly reduced in robotic nipple-sparing mastectomy (OR 0.54 (95 per cent c.i. 0.30 to 0.96); *P* = 0.03; *I*^2^ = 15 per cent). Operating time (mean difference +58.81 min (95 per cent c.i. +28.19 to +89.44 min); *P* = 0.0002) and length of stay (mean difference +1.23 days (95 per cent c.i. +0.64 to +1.81 days); *P* < 0.0001) were significantly increased in robotic nipple-sparing mastectomy, whereas the opposite was true for blood loss (mean difference −53.18 ml (95 per cent c.i. −71.78 to −34.58 ml); *P* < 0.0001).

**Conclusion:**

Whilst still in its infancy, robotic breast surgery may become a viable option in breast surgery. Nonetheless, the oncological safety of this approach requires robust assessment.

## Teaser

A total of seven studies of overall fair quality, involving 1674 patients, examining postoperative complications and operative variables that compared robotic nipple-sparing mastectomy *versus* conventional nipple-sparing mastectomy for breast cancer were included in this systematic review and meta-analysis. Overall, complication events were notably reduced in robotic nipple-sparing mastectomy, albeit not reaching statistical significance, namely grade 3 complications, re-operation, skin necrosis, seroma, and haematoma; however, postoperative infection, implant loss, and positive margins were increased in robotic nipple-sparing mastectomy. Although associated with a higher cost, a steep learning curve and patient-reported outcome measures favour robotic nipple-sparing mastectomy.

## Introduction

In 2020, 2.3 million women were diagnosed with breast cancer globally, resulting in 685 000 deaths. By the end of 2020, there were 7.8 million women alive who had been diagnosed with breast cancer in the past 5 years, making this the world’s most prevalent malignancy^1^. Remarkable advances in management have occurred in the past 30 years, including early detection, improved systemic treatments, and refined surgical approaches. An effort to improve the aesthetic outcome of breast surgery, through preservation of the nipple–areolar complex (NAC), has led to the development of nipple-sparing mastectomy (NSM)^2,3^. Initially, concerns were voiced regarding the oncological safety of NSMs due to the potential risk of local recurrence based on residual glandular tissue remaining *in situ* and occult NAC involvement^4^. Hence, the approach was initially reserved for the prophylactic treatment of women with a high risk of developing breast cancer^5^. NSMs have, however, been increasingly used also for women with breast cancer where the NAC is not involved^6–8^. Several studies have shown that disease-free survival and local recurrence rates of NSMs are equivalent to those of skin-sparing or modified radical mastectomies in selected patients^9,10^. Moreover, there is evidence that cosmetic outcomes and patient-reported outcome measures are better with NAC preservation^2,3,11,12^. In light of this evidence, there has been a steady increase in the NSM rate among women undergoing mastectomy and reconstruction due to breast cancer^13^.

A total of 15 incision types for conventional NSM (CNSM) are reported across the literature^14^, each of which has advantages and disadvantages. As an example, inframammary incisions provide limited access to the upper areas of the breast, necessitating deep retraction, use of headlights, and awkward positioning^15,16^. That said, other incisions, placed closer to the NAC, might mitigate some of these access challenges, but they may also disrupt the NAC vascular supply, thus significantly increasing the risk of necrosis^14,17–19^. Minimally invasive surgery has been introduced, along with the mainstream conventional open operations, and new surgical innovations, including endoscopic NSM (ENSM) and robotic NSM (RNSM), have emerged. Due to the limitations of endoscopic instruments and inherent technical difficulty, ENSM is not widely adopted in the surgical management of breast cancer. RNSM is also technically demanding, but it is more practical than ENSM and is therefore being more readily explored. Robotic technology employing three-dimensional imaging, high resolution, flexible instruments with greater precision, and a wider range of motion has been developed to address the limitations of endoscopic procedures. Robotic-assisted procedures have been successfully deployed in urology, gynaecology, and colorectal surgery for a variety of indications^20–24^. Toesca *et al*.^25,26^ introduced RNSM in 2017 by employing a small axillary incision to complete the resection and simultaneously performing an implant reconstruction. Since then, several groups have followed^27–29^.

In 2017, during the 15th St Gallen International Breast Cancer Conference, robotic mastectomy was recognized as an option for selected patients^30^. The US Food and Drug Administration has not approved robotic breast surgery, issuing a warning in February 2019 that the safety and efficacy of robotic devices for mastectomy have not been established^31^. Since then, several opinion papers, an international protocol, and a consensus statement have been issued^29,32–38^. All have highlighted the potential benefits and need for more evidence. While research has demonstrated the feasibility and safety of RNSM, there is a steep technical learning curve involved^22,24^. Moreover, questions remain concerning complication rates, operative variables, and, most importantly, oncological safety^24^.

The aim of this systematic review and meta-analysis was to assess complication rates, differences in operative variables, and outcomes for RNSM when compared with its mainstream counterpart (CNSM).

## Methods

A systematic review was performed in accordance with PRISMA guidelines^38^ and registered with PROSPERO, the international prospective register of systematic reviews (CRD42022381495). Five databases (Embase (Ovid), Global Health, MEDLINE (Ovid), Health Management Information Consortium (HMIC), and American Physiological Association (APA) PsycArticles) were subject to an independent literature search for relevant studies up to 13 September 2022. Additional records were not sought. The references of the included studies were scrutinized for additional relevant studies. Search limitations included human participants and English-language articles. The following search term was used in Ovid: (robotic and surgery and (breast or axill* or mastectomy or breast conserving) and breast cancer).mp.

### Inclusion and exclusion criteria

All included studies (retrospective, prospective, and time series) examined the immediate and long-term complications of RNSM. No geographical, age, or gender restrictions were applied. Studies not directly comparing RNSM with conventional nipple sparing mastectomy (CNSM) were excluded (full-text exclusion criterion).

### Data extraction

After removing duplicates, citations were screened by title and abstract, and then full texts were appraised to determine their eligibility by one author (A.N.) (*Fig. S1*). A total of two authors (A.N. and S.L.K.) independently conducted the abstract and full-text screening. Disagreements were resolved by a consensus meeting. Peer-reviewed full-text papers that reported comparison of postoperative complications, aesthetic outcome, and oncological outcomes were selected (*Table S1*). Studies were assessed for overlapping populations. Data from each article (*n* (percentage) or median (range)) were extracted by two authors (A.N. and S.L.K.): number of participants; number of participants and percentage treated with RNSM; robotic system/platform used; study interval; histology; cancer stage; indication; age (median (range)); post-menopausal status (*n* (percentage)); current smoker (*n* (percentage)); BMI (median (range)); breast size (A–B, C, and greater than C (*n* (percentage)); specimen size in grams; follow-up in months (median (range)); procedure time in minutes (median (range)); reconstruction time in minutes (median (range)); reconstruction type; incision type; length of stay in days (median (range)); and conversion to open surgery (*n* (percentage)). Where mean and standard deviation values were supplied, median values were calculated for homogenization (*Table S2*).

### Outcomes

Primary study outcomes were complications (Clavien–Dindo grade III complications, skin and nipple necrosis, wound dehiscence, infection, seroma, haematoma, and implant loss) and oncological safety (positive margins and recurrence) for RNSM compared with CNSM (*n* (percentage)). Secondary outcomes included operative variables, operating time, length of stay, blood loss, cost-effectiveness, patient satisfaction, aesthetic outcome, and learning curve.

### Quality assessment

The quality of the included studies was assessed by two independent reviewers (A.N. and S.L.K.) using the Newcastle–Ottawa scale (NOS) for observational studies^39^. Bias analysis was conducted via the Cochrane-recommended tool (Review Manager (RevMan) V. 5.4)^40^. Studies with an NOS score greater than or equal to six were of high quality. RCTs were assessed using the Cochrane RoB 2 tool^41^.

### Data analysis and meta-analysis

Clinical study context and design were compared and suitably homogeneous studies were included in the quantitative analysis ^40,42^. The meta-analysis was conducted by computing the OR or mean difference (MD) random effects from the original data using the Mantel–Haenszel method with RevMan V. 5.4 software. Statistical heterogeneity was quantified using *I*^2^ statistics and Cochrane Q tests. Asymmetry was assessed by funnel plot and publication bias was assessed formally by rank correlation test (Begg’s test); RevMan V. 5.4^41^. Given the limited number of studies meeting inclusion criteria, all studies were analysed jointly regardless of design. Clinical characteristics were employed to quantify inherent heterogeneity. Therefore, a sensitivity analysis was conducted based on median patient age across each study population, with a cut-off at 46.7 years old, which was rounded up to 47 years old.

## Results

A total of 1985 citations were initially retrieved, of which 7 studies met the inclusion criteria for full-text screening (*Fig. S1a*); 1 study was an RCT^43^, 1 study was a prospective observational study^44^, and 5 studies were retrospective observational studies^45–49^ (*Table S1*). The single RCT was considered of good quality, with a low risk of bias, as assessed using the Cochrane RoB 2 tool^41^. A total of four observational studies^44–46,49^ were deemed of overall fair quality using the NOS, whereas two observational studies were of poor quality^47,48^ (*Fig. S1b*,*c*). A total of 1674 patients, of whom 853 (50.9 per cent) underwent RNSM and 821 (49.1 per cent) underwent CNSM, were included in this systematic review and meta-analysis. All participants were female, with a median age of 46.7 (interquartile range (i.q.r.) 45.38–49.48) years and a median BMI of 22.64 (i.q.r. 21.83–32.65) kg/m^2^. Patient post-menopausal status was reported in two studies, with 39 patients (24.22 per cent of total population examined in the relevant studies) being post-menopausal at the time of treatment^46,49^. A total of 245 patients (14.6 per cent) were diagnosed with ductal carcinoma *in situ*, 444 patients (26.52 per cent) were diagnosed with invasive ductal carcinoma, and 534 patients (31.89 per cent) were diagnosed with invasive lobular carcinoma or mixed tumour pathology, while tumour histology was not stated for 26.99 per cent of the study population. Regarding cancer stage, 301 patients (17.98 per cent) were stage 0, 443 patients (26.46 per cent) were stage I, 429 patients (25.6 per cent) were stage II, 100 patients (5.97 per cent) were stage III, and 9 patients (0.53 per cent) were stage IV. The majority of RNSM procedures were performed via an axillary incision, whereas several different approaches were utilized for CNSM (*Table S3*). Considering the surgical equipment, four studies utilized the da Vinci Xi Surgical System^44–47^.

### Meta-analysis

**Fig. 1:**
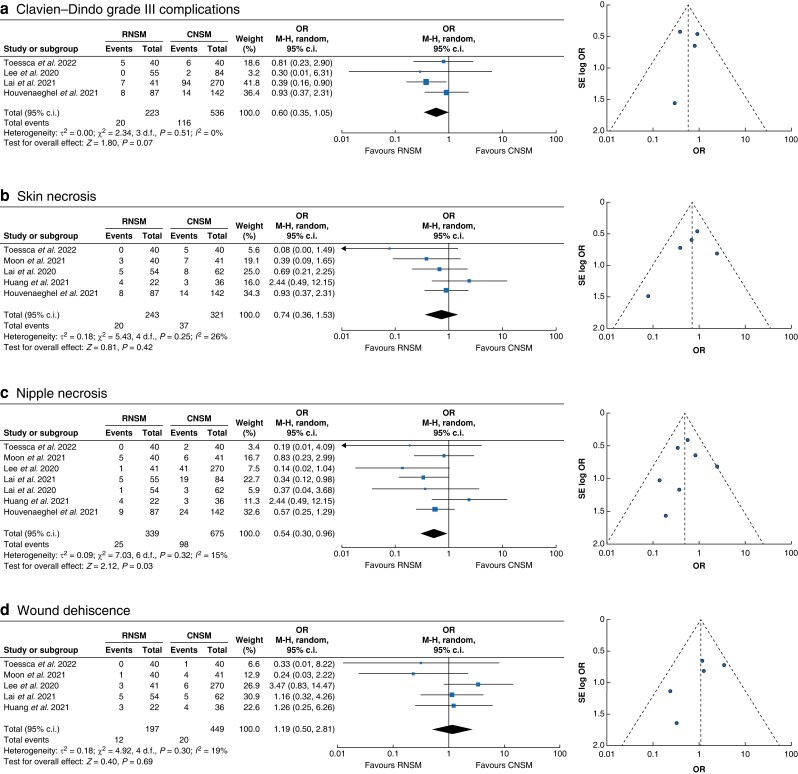
Mantel–Haenszel statistical method with random-effects analysis model and OR as output only for included observational studies and RCT, and funnel plots assessing respective variance Forest plots analysing crude event numbers between robotic nipple-sparing mastectomy and conventional nipple-sparing mastectomy. **a** Clavien–Dindo grade III complications. **b** Skin necrosis. **c** Nipple necrosis. **d** Wound dehiscence. Overall heterogeneity for the respective outcomes was considered acceptable (less than 30 per cent) given the nature of the included studies. RNSM, robotic nipple-sparing mastectomy; CNSM, conventional nipple-sparing mastectomy; M-H, Mantel–Haenszel; SE, standard error.

**Fig. 2:**
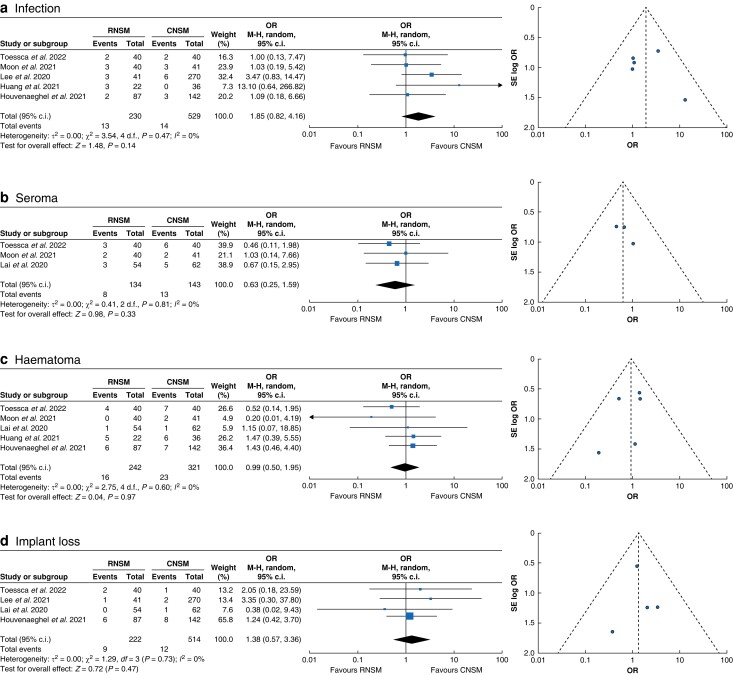
Mantel–Haenszel statistical method with random-effects analysis model and OR as output only for included observational studies and RCT, and funnel plots assessing respective variance Forest plots analysing crude event numbers between robotic nipple-sparring mastectomy and conventional nipple-sparing mastectomy. **a** Infection. **b** Seroma. **c** Haematoma. **d** Implant loss. Overall heterogeneity for the respective outcomes was considered acceptable (less than 30 per cent) given the nature of the included studies. RNSM, robotic nipple-sparing mastectomy; CNSM, conventional nipple-sparing mastectomy; M-H, Mantel–Haenszel; SE, standard error.

Overall, complications were lower in RNSM, albeit not reaching statistical significance. More specifically: Clavien–Dindo grade III complications (OR 0.60 (95 per cent c.i. 0.35 to 1.05); *P* = 0.07; *I*^2^ = 0 per cent); re-operation required (OR 0.91 (95 per cent c.i. 0.41 to 2.02); *P* = 0.51; *I*^2^ = 30 per cent); skin necrosis (OR 0.74 (95 per cent c.i. 0.36 to 1.53); *P* = 0.42; *I*^2^ = 26 per cent); seroma (OR 0.63 (95 per cent c.i. 0.25 to 1.59); *P* = 0.331; *I*^2^ = 0 per cent) and haematoma (OR 0.99 (95 per cent c.i. 0.50 to 1.95); *P* = 0.972; I² = 0 per cent) (*Fig. 1*). Nipple necrosis events were, however, significantly reduced in RNSM (OR 0.54 (95 per cent c.i. 0.30 to 0.96); *P* = 0.029; *I*^2^ = 15 per cent). There was no notable difference between RNSM and CNSM for wound dehiscence events (OR 1.19 (95 per cent c.i. 0.50 to 2.81); *P* = 0.691; *I*^2^ = 19 per cent). Whilst not statistically significant, postoperative infections appeared to be increased in the RNSM patient group (OR 1.85 (95 per cent c.i. 0.82 to 4.16); *P* = 0.138; *I*^2^ = 0 per cent), as was implant loss (OR 1.38 (95 per cent c.i. 0.57 to 3.36); *P* = 0.471; *I*^2^ = 0 per cent) (*Fig. 2*).

**Fig. 3:**
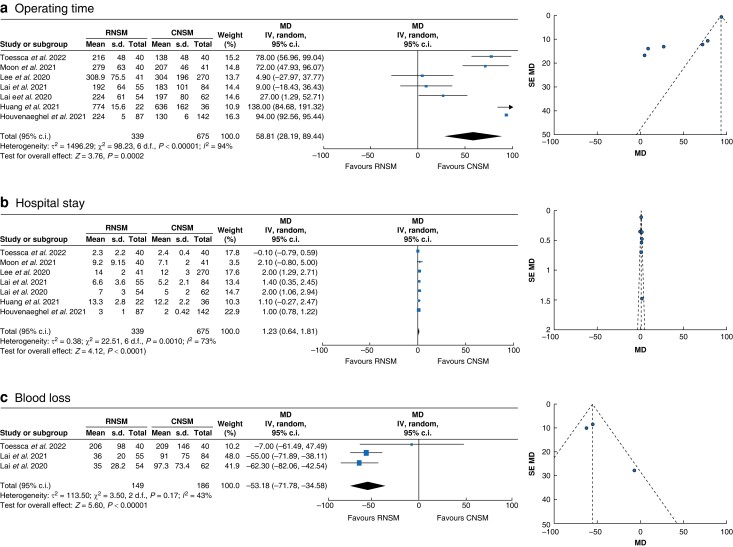
Mantel–Haenszel statistical method with random-effects analysis model and mean difference for continuous variables as output only for included observational studies and RCT, and funnel plots assessing respective variance Forest plots analysing crude event numbers between between robotic nipple-sparring mastectomy and conventional nipple-sparing mastectomy. **a** Operating time (in minutes). **b** Hospital stay (in days). **c** Blood loss (in millilitres). RNSM, robotic nipple-sparing mastectomy; CNSM, conventional nipple-sparing mastectomy; M-H, Mantel–Haenszel; SE, standard error; MD, mean difference.

Regarding oncological safety, local recurrence events appeared to be reduced in the RNSM group (OR 0.27 (95 per cent c.i. 0.07 to 1.07); *P* = 0.061; *I*^2^ = 0 per cent), whereas the opposite was observed for positive margins at surgery (OR 1.66 (95 per cent c.i. 0.46 to 5.94); *P* = 0.439; *I*^2^ = 0 per cent) (*Fig. S2*). Operating time MD (in minutes) was significantly increased in RNSM (MD +58.81 min (95 per cent c.i. +28.19 to +89.44 min); *P* < 0.001; *I*^2^ = 94 per cent). In contrast, intraoperative blood loss was significantly reduced in the RNSM group (MD −53.18 ml (95 per cent c.i. −71.78 to −34.58 ml); *P* < 0.00001; *I*^2^ = 43 per cent). A statistically significant difference favouring CNSM was noted regarding length of stay (in days) (MD +1.23 days (95 per cent c.i. +0.64 to +1.81 days); *P* < 0.001; *I*^2^ = 73 per cent) (*Fig. 3*). Importantly, the subgroup analysis for younger patients (less than 47 years old) did not show changes in complication trends compared with the total population (*Table S4*). Equally, younger patients did not show changes in operating time compared with the total population (MD 70.87 min (7.32 to 134.42 min); *P* = 0.56, *I*^2^ = 91 per cent). Therefore, younger patient age does not appear to improve length of surgery in RNSM. The learning curve, however, appeared to be strongly associated with shorter operating times (*Table S1*). Cost-effectiveness was reported by three studies, for which RNSM was a median of 48.90 (i.q.r. 34.70–52.42) per cent more expensive than CNSM^44–46^. Lastly, the aesthetic outcome was assessed in five studies, by means of patient-reported outcomes (four studies) or a panel review (one study), and was uniformly found to be better in RNSM patients^43–46^.

## Discussion

The present meta-analysis is a contemporary review of head-to-head comparisons of RNSM *versus* CNSM complications and surgical outcomes. Overall, complication rates were reduced in the RNSM group, although not reaching statistical significance for most outcomes. There was, however, a statistically significant reduction of nipple necrosis (OR 0.54 (95 per cent c.i. 0.30 to 0.96); *P* = 0.029; *I*^2^ = 15 per cent) in the RNSM group. Despite longer operations, intraoperative blood loss was significantly reduced in the RNSM group and the aesthetic outcome was deemed better in comparison with CNSM. In contrast to previously published evidence, suggesting that postoperative complications may be higher in patients undergoing RNSM (complication rate of 3.9 per cent for CNSM (total of 13 661 masectomies) *versus* complication rate of 7 per cent for RNSM (total of 225 masectomies); *P* = 0.070)^50^, the inclusion of a larger RSNM cohort (853 patients) in the present study suggests a steep upwards trend in the learning curve for RNSM. Recent observational studies also suggest an increase in RNSM surgical efficiency, although it should be noted that patient selection may significantly skew objective quantification of surgical outcomes. As an example, studies on RNSM only included patients with small to medium breast size and excluded those with breast cup sizes greater than C. Additionally, RNSM patients had a median BMI of 22.65 kg/m^2^ and the majority of those diagnosed with early-stage disease (70.04 per cent), denoting the uncertainty of RNSM performance in higher-risk patients.

Nipple necrosis is a major postoperative complication in NSMs. In RNSM, the incidence of nipple necrosis decreased significantly. Nevertheless, the definition of nipple necrosis varied among the included studies, with overlaps in the reporting of nipple necrosis, ischaemia, skin and flap necrosis. Consistent with the present study, Filipe *et al*.^50^ reported decreased skin and nipple necrosis rates in RNSM (*Table 1*). Others have reported lower nipple necrosis rates and Clavien–Dindo grade complications^51^. This is expected, as most complications after CNSM are associated with impaired blood flow. In RNSM, the incision is made away from the nipple in the mid-axillary line and the improved exposure enables the surgeon to dissect glandular tissue with greater precision, while preserving subcutaneous fat and vessels^14,17,18^. Similar rates of implant loss are recorded in the present review (4.05 per cent for RNSM and 2.33 per cent for CNSM) compared with previous reviews.

**Table 1:** Comparison of crude complication rates of robotic nipple-sparing mastectomy and conventional nipple-sparing mastectomy

Complication	RNSM		CNSM	
	Present study	Filipe *et al*.^50^	Present study	Filipe *et al*.^50^
**Implant loss**	4.05	4.1	2.33	3.2
**Haematoma**	6.58	4.3	7.16	2.0
**Necrosis**	8.23 (skin); 7.34 (nipple)	4.3	11.52 (skin); 14.22 (nipple)	7.4
**Infection**	5.56	8.3	2.64	4.0
**Seroma**	5.97	3.0	9.09	2.0

Values are %. RNSM, robotic nipple-sparing mastectomy; CNSM, conventional nipple-sparing mastectomy.

Despite previous studies reporting a high infection rate for RNSM, the present study reports a comparatively low infection rate (present study, 5.56 per cent; and Filipe *et al*.^50^, 8.3 per cent), albeit still higher than for CNSM. The opposite trend was noted for haematoma and seroma formation, favouring RNSM, although without statistical significance. The crude rates of postoperative haematoma were higher for RNSM in the present study when compared with previous studies (7.16 *versus* 4.3 per cent), but meta-synthesized evidence suggested no significant difference between RNSM and CNSM (OR 0.99 (95 per cent c.i. 0.50 to 1.95); *P* = 0.969). It is interesting to note that none of the studies reported a need of conversion to CNSM, as this likely is a rare phenomenon observed during the initial learning curve of a new technology.

Postoperative complications are of major clinical importance, but oncological safety should be the first consideration when determining whether a procedure should be performed in breast cancer. In terms of oncological safety, it appeared that there was a reduction in the number of local recurrence events in the RNSM group. Nevertheless, the follow-up duration was insufficient to draw any firm conclusions and the RNSM group is prone to selection bias. The increased risk of positive margins may reflect the inclusion of more advanced disease stages or flexibility regarding the accepted distance of the tumour to the areola. Two studies excluded disease that was greater than or equal to stage IIIb^45,46^ and one study excluded disease that was stage IV^49^ for RNSM. Toesca *et al*.^43^ included one stage IV patient in the RNSM group and Lai *et al*.^45^ included eight stage IV patients in the CNSM group. The only RCT^43^ reported on oncological outcomes and, with a median follow-up of 42 months, there was no significant difference in overall survival and disease-free survival between the RNSM and CNSM arms. Additionally, no nipple recurrence was noted in the RNSM arm, whereas one nipple recurrence was reported in the CNSM arm. Oncological outcomes were similar in the SORI study^51^. Of note, long-term oncological outcomes were not addressed in the present meta-analysis, as the oncological safety profile was not systematically reported in the included studies. For the four included studies that reported recurrence events^43,45–47^, the duration of follow-up was also deemed insufficient for reliable conclusions (*Fig. S1b*).

A learning curve is anticipated for any new technique. The increased operating time of 58 min is especially important from a health-system management point of view, as resource constrains are prevalent. Regarding the observed increased length of RNSM procedures, the time required for preparation of the operating area, docking of robotic arms, robotic breast resection, and the overall smaller operating space compared with intraperitoneal robotic surgery, and longer smoke expulsion times, may all influence the operating time. This may, however, be reduced with time. On that note, the preliminary experience and learning curve of RNSM were analysed and reported by Lai *et al*.^52,53^; the cases needed to reduce operating time for ‘docking’, ‘RNSM’, and ‘total time for RNSM and Immediate Prosthetic Breast Reconstruction (IPBR)’ were 13th, 13th, and 12th procedures respectively. Similar trends were observed in the present review, where the surgeon learning curve appeared to be strongly associated with shorter operating times (*Table S1*). A total of three of the included studies reported on the learning curve and demonstrated a steep learning curve^45–47^. The Korea Robot-Endoscopy Minimal Access Breast Surgery Study Group (KoREa-BSG)^54^ reported early experiences with 11 surgeons at eight institutions and demonstrated an equally rapid learning-curve stabilization, especially among second-generation surgeons, who learned from the pioneer surgeons.

Taking into account that the length of the operation remains high at this stage, the major drawback of RNSM is the high cost, shown to be an additional 3,804.80 Euros^46,53,54^. RNSM is significantly associated with a higher cost compared with other NSMs (*P* < 0.01)^45^. Moreover, the mean cost was reported to be higher for both RNSM-implant *versus* CNSM-implant (+34.7 per cent: 1749 euros) and RNSM-latissimus dorsi flap *versus* CNSM-latissimus dorsi flap (+30 per cent: 2357 euros) (*Table S1*)^44^. The costs of the robotic console, service contract, and disposable instrumentation are higher than the cost of a CNSM. Additional expenses were, however, minimal when approximately 300 procedures per year were performed with a single robotic system, using only two robotic instruments for dissection and a brief learning curve^28^. With an expanding range of clinical indications for the robotic approach, the overall cost of using the platform is likely to drop further. This will mainly result from a further improvement in the longevity of the instruments. Recently, the ‘life’ of each robotic instrument has increased from 10 uses to up to 18 uses, making the consumables significantly more cost-effective than previously. It is plausible that further development along the technical specifications of robotic systems may make robotic procedures more cost-effective in the longer term^55,56^. The lower postoperative complication profile of RNSM^43,51^, including reduction of postoperative complications, can moreover translate into improved long-term cost-effective outcomes.

In addition to postoperative outcomes and oncological safety, the aesthetic outcome is vital in terms of patient satisfaction and quality of life^57^. The most appealing aspect of an NSM is superior aesthetic results. The patient satisfaction or aesthetic outcome was assessed in five studies. Patient-reported outcome measures and panel-based assessment of the aesthetic outcome were reported inconsistently across studies. Toesca *et al*.^43^ documented BREAST-Q scores at 12 months after surgery and a high level of quality of life was maintained after an RNSM. Additionally, a similar trend favouring RNSM in terms of reducing psychosocial health and body-image disturbances after cancer treatment was found. It is also noteworthy that nipple sensitivity and sexual pleasure were less disturbed after the robotic approach. Houvenaeghel *et al*.^44^ examined the aesthetic outcome at 6 and 12 months after surgery using a questionnaire. Similarly, aesthetic-outcome panel questionnaires were utilized by Huang *et al*.^47^. The only study to report data on nipple sensitivity showed better preservation of nipple sensation^43^. Of note, a recent study has highlighted that patient and panel aesthetic-outcome assessments may significantly differ and therefore standardization of tools may be necessary to allow meaningful comparisons across studies^57^.

The present meta-analysis is a contemporary head-to-head comparison of RNSM *versus* CNSM complications and surgical outcomes, with a robust search strategy and use of Cochrane-recommended statistical methodology, in contrast to a previously published meta-synthesis^58^. The present study has evaluated cost-effectiveness and the learning curve, in addition to postoperative complications and outcomes. Inherent limitations of the present analysis lie in the retrospective observational design of some of the included studies, which leads to a possible risk of selection bias. The level of the evidence of the included studies is not of high quality, indicating the need of further high-quality studies. On that note, the MAstectomy with Reconstruction including Robotic Endoscopic Surgery (MARRES) study (NCT04585074) is a prospective cohort study aiming to recruit 2000 patients and evaluates surgical outcomes and complication rates between RNSM *versus* endoscopic mastectomy *versus* CNSM^59^. Additionally, the ROM trial (NCT05490433) aims to provide prospective results to address long-term oncological outcomes, including 5-year survival rates, as well as postoperative complication rates and cost-effectiveness of RNSM^60^. Contemporary and recently completed clinical trials associated with robotic breast surgery have been thoroughly summarized by Park *et al*.^61^. Multiple prospective trials have been initiated on RNSM and will provide higher level of evidence^62–72^.

Additionally, while the aim of the present study was to evaluate the outcomes of these operations on women with breast cancer, the included studies were conducted on very heterogeneous patient populations, including women undergoing both risk-reducing mastectomies, as well as cancer treatment. For data transparency and homogenization of cumulative outcomes, crude outcome values indication for surgery, disease stage (*Table S2*), and surgical parameters (*Table S3*) were collected and reported. Notably, the included studies utilized a variety of reconstruction techniques and it is well established that the complication profile of autologous reconstruction may differ according to the technology used. Neoadjuvant and adjuvant therapy may also influence outcomes; however, this variable was not reported uniformly in the included studies and hence was not incorporated in the meta-analysis. Of note, the proportion of invasive lobular carcinoma or mixed tumour pathology (31.89 per cent) appeared higher than expected, especially when compared with the 26.52 per cent of invasive ductal carcinoma. A likely cause could lie in lobular cancer more often being multifocal, requiring a higher rate of mastectomy, as well as geographical variations in invasive lobular carcinoma incidence.

Of note, most studies in the present study were conducted in Taiwan and Korea, limiting the generalizability of the results to Western populations. However, in selected patients, RNSM has reduced or equivalent postoperative complications and oncological safety. Overall, the present systematic review and meta-analysis indicates a trend of reduced complication rates and improved aesthetic outcome in patients that undergo RNSM. Cost-effectiveness and learning curve-dependent longer operating times pose limitations to widespread adoption. Whilst still in its infancy, robotic breast surgery may present a viable option within the range of oncoplastic breast-surgery techniques. The oncological safety profile of this approach, however, requires more robust assessment before its widespread implementation.

## Supplementary Material

znad336_Supplementary_Data

## Data Availability

All crude data available upon request.
