# Supplementary material for: Postoperative complications and surgical outcomes of robotic versus conventional nipple-sparing mastectomy in breast cancer: meta-analysis
Source: Br J Surg. 2023 Oct 27;111(1):znad336. doi: 10.1093/bjs/znad336 (PMC10769157; doi:10.1093/bjs/znad336)
Supplement: znad336_Supplementary_Data [file znad336_supplementary_data.zip › supplementary-material 280923.docx]

**Post-operative complications and surgical outcomes of robotic vs. conventional nipple sparing mastectomy in breast cancer: Systematic review and meta-analysis.**

**Nessa A.^1,2,3^, Shaikh S.^1,2^, Fuller M.^3^, Masannat Y.A.^1,3^, Kastora S.L.^4^**

**^1.^ School of Medicine, Medical Sciences and Nutrition, University of Aberdeen, Aberdeen, UK.**

**^2.^ Aberdeen Royal Infirmary, General Surgery, Foresterhill, Aberdeen, UK.**

**^3.^ Aberdeen Royal Infirmary, Breast Surgery, Clinic E, Foresterhill, Aberdeen, UK.**

**^4.^ University College London, UCL EGA Institute for Women's Health, Medical School Building, 74 Huntley Street, London, UK**

**Corresponding author:**

**Ashrafun Nessa MBBS, FCPS, MRCS(Eng), PG Cert (General Surgery)**

**Honorary Associate, University of Aberdeen**

**Specialty Registrar year 6, General Surgery**

**North of Scotland, NHS Grampian, Aberdeen Royal Infirmary**

**Address: 9 weaver Terrace, Aberdeen, AB24 4SD, United Kingdom**

**Email: ashrafun.nessa@abdn.ac.uk;** [**ashrafun.nessa@nhs.scot**](mailto:ashrafun.nessa@nhs.scot)**;** [**drashrafunnesa@hotmail.com**](mailto:drashrafunnesa@hotmail.com)

| **Supplementary Figures and Tables** |  |
| --- | --- |
| **Supplementary Figure 1**  PRISMA diagram and risk of bias assessment. (A) PRSIMA Flow chart (B) Risk of Bias Summary (C) Risk of Bias Graph, per Newcastle-Ottawa Scale, summative percentages across all included studies. | *page 3* |
| **Supplementary Figure 2**  Mantel-Haensel statistical method with random effects analysis model and odds ratio (A,B) as output only for included observational studies and for RCTs and funnel plot assessing respective variance. Respective forest plots analysing crude event numbers between RNSM vs CNSM for the following outcomes A. Positive Margins B. Local Recurrence | *page 4* |
| **Supplementary Table 1**  PICO Chart: RNSM Robotic nipple sparing mastectomy; CSNM Conventional nipple sparing mastectomy; NAC Nipple-areolar complex; NACT Neoadjuvant chemotherapy, IBC Invasive breast cancer; DCIS Ductal cancer in situ; BCS Breast conserving surgery | *page 5* |
| **Supplementary Table 2**  Clinical and demographic population characteristics. Values are n (%) unless otherwise indicated’ | *page 8* |
| **Supplementary Table 3**  Operative characteristics. Values are n (%) unless otherwise indicated. SLNB: Sentinel Lymph node biopsy; RNSM Robotic nipple sparing mastectomy; CSNM Conventional nipple sparing mastectomy | *page 10* |
| **Supplementary Table 4**  Sensitivity analysis. Age cut-off <47 (Median age across all groups) | *page 14* |
|  |  |
|  |  |
|  |  |
|  |  |
|  |  |
|  |  |
|  |  |

**Supplementary Figures and Tables**

**Supplementary Figure 1**


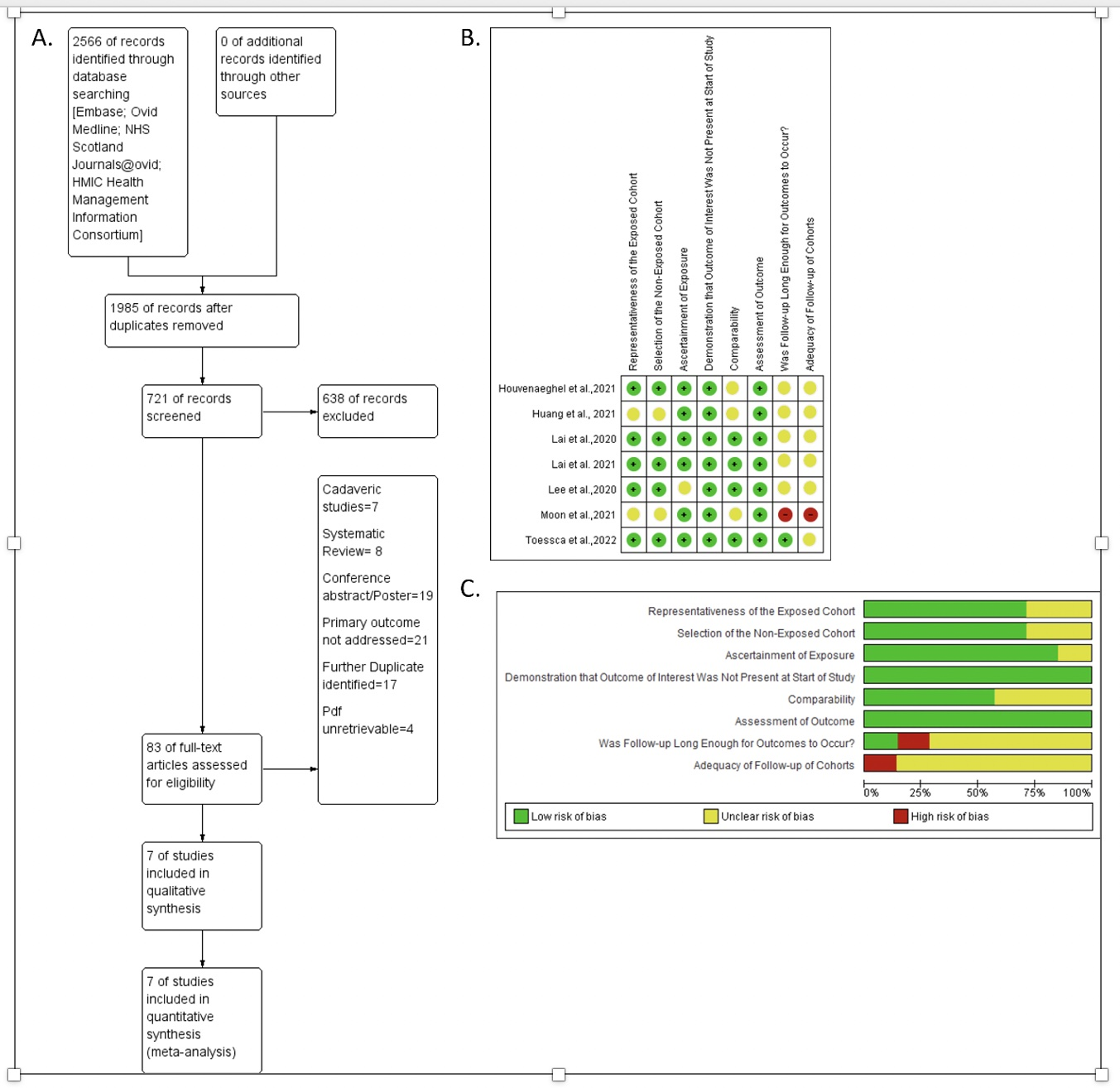


**Supplementary Figure 1**

PRISMA diagram and risk of bias assessment. (A) PRSIMA Flow chart (B) Risk of Bias Summary (C) Risk of Bias Graph, per Newcastle-Ottawa Scale, summative percentages across all included studies.

**Supplementary Figure 2**


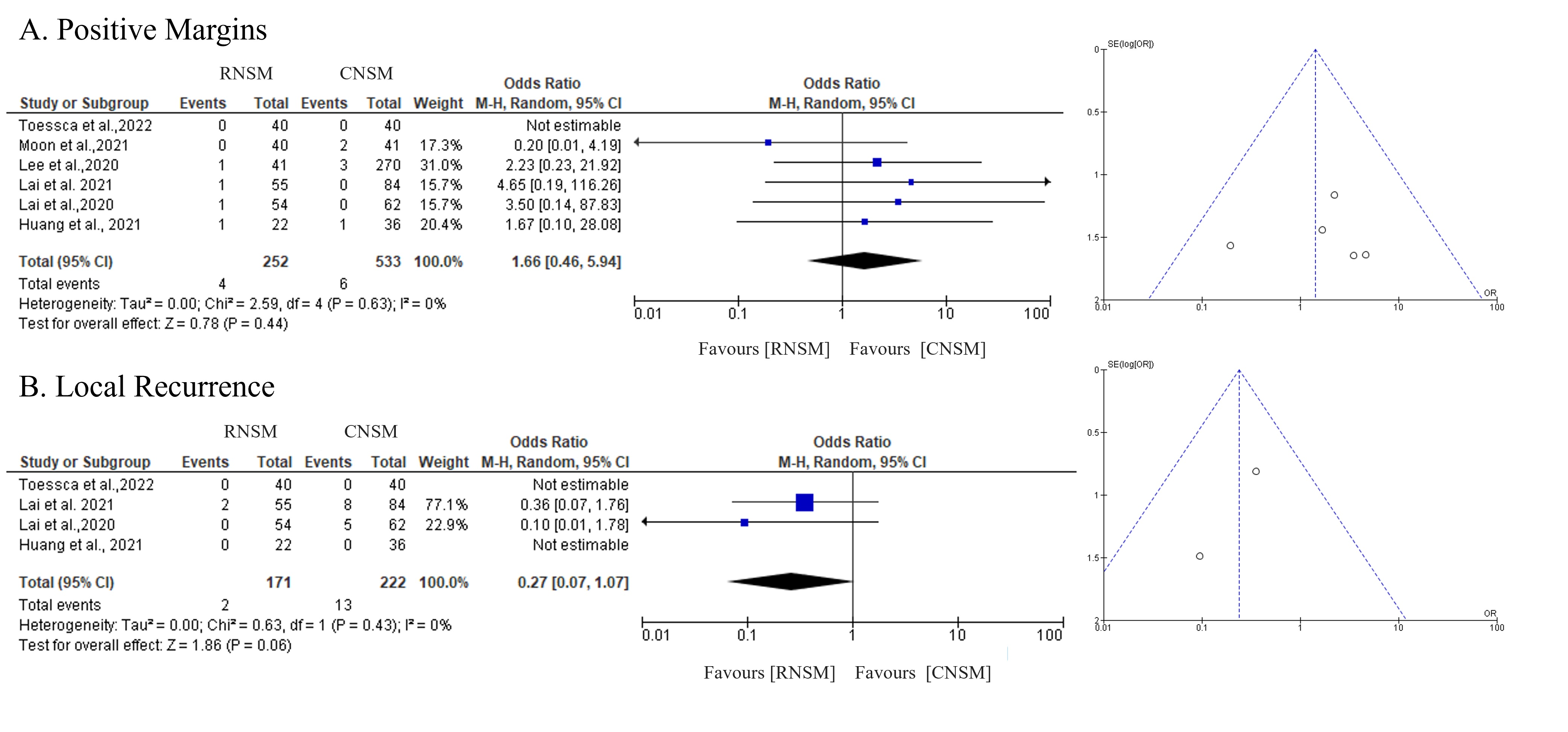


**Supplementary Figure 2**

Mantel-Haensel statistical method with random effects analysis model and odds ratio (A,B) as output only for included observational studies and for RCTs and funnel plot assessing respective variance. Respective forest plots analysing crude event numbers between RNSM vs CNSM for the following outcomes A. Positive Margins B. Local Recurrence

**Supplementary Table 1: PICO Chart;** RNSM Robotic nipple sparing mastectomy; CSNM Conventional nipple sparing mastectomy; NAC Nipple-areolar complex; NACT Neoadjuvant chemotherapy, IBC Invasive breast cancer; DCIS Ductal cancer in situ; BCS Breast conserving surgery

| First author | Study Type | Country | Total population | Learning curve | Aesthetic Outcome | Cost effectiveness | Inclusion criteria | Exclusion criteria | Outcome (s) | Conversion required(n%) | Interpe  tation |
| --- | --- | --- | --- | --- | --- | --- | --- | --- | --- | --- | --- |
| Toesca et al. 2022 | RCT (NCT03440398) | Italy | 80 | NA | Breast Q score at 12 months RNSM 70.9±21.2; CNSM 47.3±21.6 | NA | IDC, DCIS or genetic predisposition (pathogenic BRCA1 or BRCA2 mutation), aged 18 years or older, candidates for nipple-sparing mastectomy with immediate breast reconstruction. Multifocal and multicentric cancers were allowed as well as any clinical tumour size, with tumours >1 cm from the NAC on clinical examination and breast imaging. | Preoperative axillary lymph node metastasis, inflammatory breast cancer, tumour involvement in skin or NAC, Pagets’ disease, mesenchymal or recurrent breast cancer, history of previous thoracic radiation therapy, pregnant, ASA score >2, uncontrolled diabetes mellitus, prior or current heavy smoking (>20ciga- cigarettes/day), large breast volume (>cup D) or previous ipsilateral breast surgery. | Surgical complications, QoL, oncologic safety | 0 (0%) | Favours RNSM  for all outcomes |
| Huang et al. 2021 | Retrospective case series | Taiwan | 63 | Non-significant trend of reduced time of mastectomy, reconstruction, and total surgery. | Panel-based aesthetic outcome superior in the RNSM | NA | NA | NA | Surgical complications, aesthetic outcome, oncologic safety | 0 (0%) | Favours RNSM  for all outcomes |
| Lai et al. 2021 | Retrospective | Taiwan | 824 | As technique matures, the operation time decreased and eventually merged without apparent difference  (p=0.12) | PROs:  RNSM (92% excellent, 8% good) CNSM (75.6% excellent, 24.4% good) | RNSM higher cost, by 4,000 USD | Early-stage breast cancer (DCIS, stage I-IIIA), tumour size <3 cm (BCS) or not >5 cm (mastectomy), absence of apparent lymph nodes metastases, skin or chest wall invasion. | Inflammatory breast cancer, chest wall or skin invasion, locally advanced breast cancer, extensive axillary lymph node metastasis (stage IIIB or later), and severe comorbidities (cardiac disease, renal failure, liver dysfunction, and poor performance status). Grade 3 ptosis of nipple was excluded from RNSM | Surgical complications, aesthetic outcome, oncologic safety, cost-effectiveness | 0 (0%) | Favours RNSM  for all outcomes except cost-effectiveness |
| Moon et al. 2021 | Retrospective | Korea | 81 | NA | NA | NA | Unilateral NSM and prepectoral implant-based reconstruction | Patients with previous breast surgery, other simultaneous surgeries, or could not be administered intravenous patient-controlled analgesia for postoperative pain control | Surgical complications, aesthetic outcome, oncologic safety. | 0 (0%) | Favours RNSM  for all outcomes |
| Houvenaeghel et al. 2021 | Prospective | France | 229 | NA | PROs:  RNSM (64.7% very good/good); CSNM (50.7% very good/good) | Mean cost was higher (+34.7%; 1749 Eur) for RNSM | Prophylactic mastectomy, local recurrence when a second BCS was not possible or not the patient’s choice, and primary BC with indication for mastectomy. | NA | Surgical complications, aesthetic outcome, oncologic safety, cost-effectiveness | 0 (0%) | Favours RNSM  for all outcomes except cost-effectiveness |
| Lee et al. 2020 | Case –control study | Korea | 281 | Mean RNSM time was 181.5 min, and longer than mastectomy time CNSM group (95.5 min).  RNSM operation times decreased over time, a significant learning curve | NA | NA | NA | Stage IV breast cancer, NACT, and previous breast surgery or radiation therapy. | Surgical complications, oncologic safety, operative time, hospital stay | 0 (0%) | Favours RNSM for surgical complications, oncologic safety. Favours CNSM for operative time, hospital stay. |
| Lai et al. 2020 | Case–control study | Taiwan | 116 | RNSM time decreased from 287 ± 77 mins (case #1–12) to 236 ± 31 mins (case #13–26, P = 0.02) | PROs: RNSM: (92% excellent, 8% good); CNSM (75.6% excellent, 24.4% good) | RNSM cost higher (10,877 ± 796 versus CNSM 5702 ± 661 USD, P<0.01). | Early stage breast cancer (DCIS, stage I - IIIA), tumour size <5cm, not extensive lymph nodes metastases, no nipple, skin, or chest wall in- vasion. For RNSM patients with small to medium sized, none to mildly ptotic breast, breast cup size C and below. | NAC involvement, inflammatory breast cancer, chest wall or skin invasion, locally advanced breast cancer, extensive axillary lymph nodes metastases (stage IIIB or later), and patients with severe co-morbid conditions (heart disease, renal failure, liver dysfunction, and poor performance status), breast cup size >E or breast mastectomy weight >600g and ptotic breast excluded from RNSM. | Surgical complications, aesthetic outcome, oncologic safety, cost-effectiveness | 0 (0%) | Favours RNSM  for all outcomes except cost-effectiveness |

**Supplementary Table 2** Clinical and demographic population characteristics. Values are n (%) unless otherwise indicated’.

| First author | Study Type | Country | Total population | % Robotic | Study period | Histology | Stage  N | Median age year (Range) | Post-menopausal status: N (%) | Current Smoker N(%) | BMI (Median, Range) | Median follow up time months, (range) |
| --- | --- | --- | --- | --- | --- | --- | --- | --- | --- | --- | --- | --- |
| Toesca et al. 2022 | RCT (NCT03440398) | Italy | 80 | 50% | 2017 to 2018 | DCIS 12 (15%);  IDC 57 (71.25%); ILC/Mixed 0 (0%) | Stage 0= 12;  Stage I=27;  Stage II=18;  Stage III=2;  Stage IV=1 | 45 (29–62) | 15 (18.75) | 7  (8.75) | NA | 28.6 (3.7-43.3) |
| Huang et al. 2021 | Retrospective case series | Taiwan | 63 | 34.90% | 2018 to 2020 | DCIS 18 (28.5%); IDC 40 (63.5%);  ILC/Mixed 2 (3.17%) | Stage 0= 28; Stage I=14; Stage II=25; Stage III=6; Stage IV=0 | 45.5 (34.7–53.6) | NA | 1 (1.58) | 35.85 (19.5-27) | 14.07 (11.34 to 15.9) |
| Lai et al. 2021 | Retrospective comparative | Taiwan | 824 | 69.10% | 2011 to 2020 | DCIS 143(17.6%);  IDC 137 (16.9%)  ILC/Mixed 532 (64.6%) | Stage 0=139; Stage I=227; Stage II=285; Stage III=71; Stage IV=8 | 50.9 (40.3-60.5) | NA | NA | NA | 49.9 (29.9- 81.7) |
| Moon et al. 2021 | Retrospective | Korea | 81 | 49.30% | 2018 to 2020 | Not stated | Stage 0= 2; Stage I=49; Stage II=15; Stage III=0; Stage IV=0 | 47.5 (38–59) | 24 (29.6) | 2; (2.46) | 23.05 (18.7-27.5) | 3 |
| Houvenaeghel et al. 2021 | Prospective | France | 229 | 37.90% | 2016 to 2020 | NA | NA | NA | NA | 50; (21.84) | 21.7 (21.1-22.9) | 1 |
| Lee et al. 2020 | Case–control study | Korea | 281 | 14.59% | 2016 to 2019 | DCIS 72 (25.6%); IDC;210 (74.7%) ILC/Mixed 0 (0%) | Stage 0= 76; Stage I=146; Stage II=53; Stage III=7; Stage IV=0 | 45.93 (37.6-54.27) | NA | Not stated | 22.23 (19.4-25.6) | 14.5 (1–28) |
| Lai et al. 2020 | Case–control study | Taiwan | 116 | 46.50% | 2011 to 2019 | NA | Stage 0= 22; Stage I=29; Stage II=33; Stage III=14; Stage IV=0 | 49  (39-59) | NA | NA | NA | 32.1 (9.5-54.7) |

**Supplementary Table 3** Operative characteristics. Values are n (%) unless otherwise indicated. SLNB: Sentinel Lymph node biopsy; RNSM Robotic nipple sparing mastectomy; CSNM Conventional nipple sparing mastectomy

| First author | Total population | Indication n (%) | Axillary procedure n (%) | System or Platform used | Pressure mmHg | Incision  n(%) | Type of reconstruction | Breast size A-B , C, >C n, (%) | Specimen size (median) g | Median follow up time (months, range) | Procedure time (mins) | Reconstruction time (mins) | Length of hospital stay (days) | Conversion |
| --- | --- | --- | --- | --- | --- | --- | --- | --- | --- | --- | --- | --- | --- | --- |
| Toesca et al. 2022 | 80 | i.Risk reducing surgery RNSM 6(15) CNSM 5 (12.5) ii. DCIS RNSM 7 (17.5) CNSM 5 (12.5). iii.Invasive BC RNSM 27(67.5) CNSM 30(75.0) | i. No axilla RNSM 6(15) CNSM 5 (12.5).  ii. SLNB RNSM 27(67.5) CNSM 28 (70.0)  iii. ALND RNSM 7 (17.5) CNSM 7 (17.5) | da Vinci Xi Surgical System | NA | i. Open radial external RNSM 0 (0.00) CNSM 34 (85), ii. periareolar RNSM 0 (0.0) CNSM 3(7.5), iii. periareolar with radial internal extension RNSM 0 (0.0) CNSM 1(2.5), iv. periareolar with radial external extension RNSM 0 (0.0) CNSM 1(2.5), v. in the inframammary fold RNSM 0 (0.0) CNSM 1(2.5),  vi. Axillary RNSM 22 (100.0)  CNSM 0 (0.0) | Retropectoral immediate prosthesis breast reconstruction (IPBR)  Using direct-to-implant (DTI) or tissue expander | A= 20 (25%);  B=43 (53.75%); C= 12 (15%); D and Over=2 (2.5%) | NA | 28.6 (3.7-43.3) | RNSM 216 ±48; CNSM 138 ±48 | RNSM 84±3; CNSM 66±36 | RNSM: 2.3±1.2; CNSM 2.4±0.6 | 0 (0%) |
| Huang et al. 2021 | 63 | i. DCIS RNSM 4 (18.2) CNSM 14 (34.1) ii. Invasive RNSM 18 (81.1) CNSM 27 (65.8) | NA | Intuitive Xi system | NA | i. Peri-areolar RNSM 0 (0.0); CNSM 4 (9.8)  ii. Inframammary fold RNSM 0 (0.0); CNSM 5 (12.2)  iii. Anterior axillary line RNSM 22 (100.0) CNSM 13 (31.7) iv. Others in anterior breast RNSM 0 (0.0) CNSM 19 (46.3) | Immediate microsurgical breast reconstruction using perforator flaps | NA | RNSM 350.4 ± 158.1; CNSM 361.4 ± 165.0 | 14.07 (11.34 to 15.9) | RNSM 774 ± 15.6; CNSM 636 ± 162 | NA | RNSM 13.3 ± 3.3; CNSM 12.2 ± 2 | 0 (0%) |
| Lai et al. 2021 | 824 |  |  | da Vinci Xi Surgical System | Not stated | NA | NA | NA | RNSM 309+- 125; CNSM 401+-343 | 49.9 (29.9- 81.7) | RNSM 192 ± 64; CNSM: 183 ± 101 | NA | RNSM: 6.6 ± 1.6; CNSM 5.2 ± 1.7 | 0 (0%) |
| Moon et al. 2021 | 81 |  | i.SLNB RNSM 37(93%) CNSM 36(88%). Ii.ALNB. 3 (8%) CNSM 5(12%) | da Vinci SP surgical system | 8-10 mm | RNSM axillary 40 (100); CNSM 41(100) inframammary, radial, and/or periareolar/ circumareolar incision  (numbers not stated) | Prepectoral immediate implant breast reconstructionusing direct-to-implant or tissue expander | NA | RNSM 388.7 ± 169.5; CNSM 421.4 ± 176.0 | 3 | RNSM 279 ± 63; CNSM: 207 ± 46 | NA | RNSM: 9.2 ± 2.7; CNSM: 7.1 ± 2.0 | 0 (0%) |
| Houvenaeghel et al. 2021 | 229 | i. Primary RNSM 70(80.5); CNSM 78(52.8)  ii. Local recur RNSM 10 (11.5) CNSM 7(4.9)  iii. Risk reducing  RNSM 7(8.5) CNSM 60( 42.3) | i. SLNB RNSM 37(42.5%) CNSM 59(41.5%)  ii. ALND RNSM 17(19.5%) CNSM 5(3.5%) | da Vinci Surgical System | 7 mm | i. peripheric RNSM 83 (95.4;) CNSM 54/(38.0) ii. areolar/radial RNSM 1 (1.1) CNSM 64 (5.1);  iii. Previous incision RNSM 3(4) CNSM 13 (9.2) iv. inversed T 1 RNSM 0/(0 CNSM 11/(7.7) | Immediate breast reconstructionwith implant or with LDF | A-B = 142 (62.0%); C=58 ( 25.3%); D=30 (13.1%) | RNSM 377(346-426);  CNSM 236(250-309) | 1 | RNSM 224± 12.3; CNSM 130 ± 16 | NA | RNSM 3± 2.9; CNSM 2±2.18 | 0 (0%) |
| Lee et al. 2020 | 281 | i. Benign CNSM 5 (1.9) RNSM 4 (9.8).  ii. DCIS CNSM 63 (23.3) RNSM 9 (22.0) ii. invasive BC  CNSM 185 (68.5). RNSM 5 (61.0) iv. BRCA mutation carrier CNSM 17 (6.3) RNSM 3 (7.3) | i. SLNB RNSM 32 (94.1) CNSM 239 (92.3) ii.ALND  3 (8.8) CNSM 35 (13.5) | da Vinci Xi Surgical System | NA | i. IMF RNSM 0 (0.0). CNSM 2 (0.7)  ii. Radial. RNSM 0 (0.0). CNSM 51 (18.9)  iii. Upper-periareolar with extension RNSM 0 (0.0) CNSM 32 (11.9),  iv. Lower-periareolar with extension RNSM 0 (0.0) CNSM 52 (19.3).  v. Circumareolar RNSM 0 (0.0). CNSM 3 (1.1) vi. Elliptical. RNSM 0 (0) CNSM 12 (4.4) vii.Lateral or axillary RNSM 41 (100.0). CNSM (0.) |  | NA |  | 14.5 (1–28) | RNSM 308.9 ±75.5; CNSM 303.9±195.9 | RNSM 140.5± 52.5; CNSM 196.8±182.5 | RNSM 14±4.; CNSM 12±3 | 0 (0%) |
| Lai et al. 2020 | 116 | Primary operable breast cancer | NA | da Vinci Xi Surgical System | 8=10mm | Not Stated | Nipple sparing mastectomy and immediate implant breast reconstruction | NA | RNSM 293 ± 113; CNSM 321.5 ± 314 | 32.1 (9.5-54.7) | RNSM 224±61; CNSM 197 ± 79.9 | NA | RNSM | 0 (0%) |

**Supplementary Table 4** Sensitivity analysis. Age cut-off <47 (Median age across all groups)

**
